# Randomized phase II trial of lymphodepletion plus adoptive cell transfer of tumor-infiltrating lymphocytes, with or without dendritic cell vaccination, in patients with metastatic melanoma

**DOI:** 10.1136/jitc-2021-002449

**Published:** 2021-05-21

**Authors:** Chantal Saberian, Rodabe N. Amaria, Amer M. Najjar, Laszlo G. Radvanyi, Cara L. Haymaker, Marie-Andrée Forget, Roland L. Bassett, Silvana C. Faria, Isabella C. Glitza, Enrique Alvarez, Sapna Parshottam, Victor Prieto, Gregory Lizée, Michael K. Wong, Jennifer L. McQuade, Adi Diab, Cassian Yee, Hussein A. Tawbi, Sapna Patel, Elizabeth J. Shpall, Michael A. Davies, Patrick Hwu, Chantale Bernatchez

**Affiliations:** 1 Melanoma Medical Onoclogy, The University of Texas MD Anderson Cancer Center, Houston, Texas, USA; 2 Department of Pediatrics - Research, Division of Pediatrics, The University of Texas MD Anderson Cancer Center, Houston, TX, USA; 3 Ontario Institute for Cancer Research, Ontario, Ontario, Canada; 4 Department of Translational Molecular Pathology, The University of Texas MD Anderson Cancer Center, Houston, TX, USA, Houston, TX, USA; 5 Department of Biostatistics, The University of Texas MD Anderson Cancer Center, Houston, Texas, USA; 6 Department of Radiology, The University of Texas MD Anderson Cancer Center, Houston, Texas, USA; 7 Department of Stem Cell Transplantation and Cellular Therapy, The University of Texas MD Anderson Cancer Center, Houston, Texas, USA; 8 Department of Biologics Development, The University of Texas MD Anderson Cancer Center, Houston, Texas, USA; 9 Department of Pathology, The University of Texas MD Anderson Cancer Center, Houston, Texas, USA

**Keywords:** adaptive immunity, dendritic cells, lymphocytes, tumor-infiltrating, melanoma, vaccination

## Abstract

**Background:**

The adoptive transfer of tumor-infiltrating lymphocytes (TIL) has demonstrated robust efficacy in metastatic melanoma patients. Tumor antigen–loaded dendritic cells (DCs) are believed to optimally activate antigen-specific T lymphocytes. We hypothesized that the combined transfer of TIL, containing a melanoma antigen recognized by T cells 1 (MART-1) specific population, with MART-1-pulsed DC will result in enhanced proliferation and prolonged survival of transferred MART-1 specific T cells in vivo ultimately leading to improved clinical responses.

**Design:**

We tested the combination of TIL and DC in a phase II clinical trial of patients with advanced stage IV melanoma. HLA-A0201 patients whose early TIL cultures demonstrated reactivity to MART-1 peptide were randomly assigned to receive TIL alone or TIL +DC pulsed with MART-1 peptide. The primary endpoint was to evaluate the persistence of MART-1 TIL in the two arms. Secondary endpoints were to evaluate clinical response and survival.

**Results:**

Ten patients were given TIL alone while eight patients received TIL+DC vaccine. Infused MART-1 reactive CD8^+^ TIL were tracked in the blood over time by flow cytometry and results show good persistence in both arms, with no difference in the persistence of MART-1 between the two arms. The objective response rate was 30% (3/10) in the TIL arm and 50% (4/8) in the TIL+DC arm. All treatments were well tolerated.

**Conclusions:**

The combination of TIL +DC showed no difference in the persistence of MART-1 TIL compared with TIL therapy alone. Although more patients showed a clinical response to TIL+DC therapy, this study was not powered to resolve differences between groups.

**Trial registration number:**

NCT00338377.

## Introduction

Adoptive cell therapy (ACT) using autologous tumor-infiltrating lymphocytes (TIL) has demonstrated high response rates in patients with metastatic melanoma and has shown activity in other histologies.^1–7^ In ACT protocols, patients are given preparative non-myeloablative lympho-depleting chemotherapy regimens before the infusion of ex vivo-expanded autologous TILs, followed by high-dose interleukin-2 (IL-2).^8^ This approach has resulted in objective response rates of 38% to over 50% in patients with metastatic melanoma, with durable, complete tumor regression observed in up to 20% of patients.^1 2 8–13^ Persistence of the transferred TIL has been associated with clinical response in early trials.^9 14^ Combinatorial approaches to improve on TIL therapy are currently being developed and tested in clinical trials. Important for the sustained antitumor activity of TIL is their proper reactivation in vivo, once the TIL migrate back to the tumor. Although checkpoint blockade can sustain the function of an already activated T-cell, full antigenic presentation by a professional antigen-presenting cell such as a dendritic cell (DC) would provide a more powerful and comprehensive activation allowing full effector function.^15^ As such, the presence of DC at the tumor site has been shown to play an important role in the response to cancer immunotherapy, including to the adoptive transfer of antitumor T cells.^16^

The use of autologous tumor antigen loaded DC as cancer vaccine has generated promising data. DCs are professional antigen-presenting cells that can efficiently activate T lymphocytes.^17 18^ Therefore, DCs may be used as clinical reagents to induce antitumor immunity by activating tumor-specific T lymphocytes in vivo that can mediate tumor destruction.^19 20^ DCs can be loaded with tumor antigens in various ways to enhance the presentation of antigen to tumor antigen-specific T cells, including pulsing DCs with peptides, proteins and whole tumor cell lysates; transducing DCs with viral vectors that express tumor antigens; expressing tumor-derived messenger RNA in DCs; and fusing DCs with tumor cells.^21^ Melanoma TIL consist of a heterogenous population of T cells enriched for tumor recognition. Although T cells recognizing patient-specific mutations are generally found in the tumor, TIL recognizing tumor antigens shared between patients and aberrantly expressed by the tumor, such as melanoma antigen recognized by T cells 1 (MART-1), gp100, and tyrosinase, are also prevalent.^22 23^ Preclinical mouse modeling demonstrated the extended persistence of adoptively transferred gp100-reactive T cells when combined with antigen-specific DC vaccination resulting in superior tumor control.^24^

Several clinical trials are currently in progress using antigen-loaded DCs with other immunotherapeutic therapies to immunize cancer patients.^25–27^ In a phase I trial of 39 patients with pretreated advanced melanoma who were treated with ipilimumab (10 mg/kg) in combination with DC vaccination, 15 patients (38%) experienced an objective tumor response rate.^26^ In a phase I clinical trial of 16 patients with melanoma who were treated with the combination of MART-1 peptide-pulsed DCs and tremelimumab, four (25%) had durable objective tumor responses that were higher than what was expected from each agent alone.^28^ Interestingly, the combination of the DC vaccine and ACT has been tested in a very limited number of patients.^29 30^ A pilot phase I clinical trial first demonstrated that the combination of the whole-tumor lysate DC vaccine and TIL was safe and feasible in eight patients with melanoma.^29^ More recently, a phase I trial investigated the safety and feasibility of TIL and DC vaccination in patients with advanced melanoma experiencing progression on immune checkpoint inhibitors and four of the four (4/4) patients had objective response rate.^30^

To capitalize on the power of DC to properly and effectively activate T cell, we hypothesized that the combination of TIL with antigen-pulsed DCs will result in enhanced proliferation and prolonged survival of transferred antigen-specific T cells in vivo and more efficient trafficking to tumor sites, ultimately leading to improved clinical responses. We chose MART-1 as a model antigen for its widespread expression in melanoma and the high frequency of MART-1 reactive T cells in the blood and tumor of melanoma patients. We evaluated the combination of TIL containing a population of MART-1 reactive T cells and MART-1 pulsed DC vaccine versus TILs alone in a randomized phase II clinical trial in patients with advanced melanoma.

## Patients and methods

### Study approval and design

The US Food and Drug Administration and the Institutional Review Board at The University of Texas MD Anderson Cancer Center (MDACC) (Houston, Texas) approved the study. The MDACC Investigational New Drugs Office performed study monitoring. This study was conducted according to the principles of the Declaration of Helsinki. All study participants granted written informed consent prior to treatment initiation.

In this randomized phase II trial, patients with metastatic melanoma underwent lympho-depleting non-myeloablative chemotherapy, followed by infusion of autologous tumor derived TIL, with or without autologous DC. Patients were randomly assigned to receive TIL alone or TIL plus DC pulsed with MART-1 peptide (MART-126-35(27L).

### Patient selection

Patients aged ≥12 years with locally advanced stage III or IV cutaneous, mucosal, or uveal melanoma were eligible for this study. The trial was organized in two turnstiles. Patients first consented to Turnstile 1 which enabled tumor resection for TIL expansion. Following expansion of adequate TIL numbers, patients were offered to consent for Turnstile 2 of the study to receive therapy. Eligibility for Turnstile 2 (treatment) included being HLA-A0201 positive, the detection of over 0.1% MART-1-reactive CD8^+^ T cells in the expanded TIL product and having measurable disease after the biopsy for TIL harvest per Immune Response Criteria (irRC) in Solid Tumors. Eligible patients had an Eastern Cooperative Group (ECOG) Performance Status of 0 to 2 and adequate bone marrow, hepatic, and renal function. Patients who had been previously treated with surgery, radiotherapy, chemotherapy, targeted therapy, and immunotherapy were eligible, and there was no limit on the number of prior therapies. Patients with brain metastases ≤1 cm who were asymptomatic and patients with known stable brain metastases were enrolled. The exclusion criteria included any medical history of cardiovascular or respiratory disease or immunodeficiency. For additional criteria, refer to clinical trial NCT00338377 on the NCI website. This current study is a report of one of the cohorts of this clinical trial, which is a randomized study of TIL with or without DC vaccine (Cohort A).

### Study endpoints and study assessments

The primary endpoint of this randomized trial was to compare the persistence of infused TIL in patients receiving adoptively transferred TIL containing MART-1-specific T cells in combination with DC pulsed with MART-1 peptide and high-dose IL-2 with that in patients receiving TIL containing MART-1-reactive T cells and high-dose IL-2 alone. A priori, we had little historical data on persistence, so no specific cut-off was defined in the protocol. We compared the level of persistence between the arms instead of introducing arbitrary cutoffs to dichotomize or categorize response, and no sample size calculations were pre-specified for this endpoint. The secondary endpoints included tumor response and survival, and no sample size calculations were prespecified for these endpoints.

Blood samples were collected from patients at the time of surgery for TIL harvest or prior to lympho-depletion (baseline), as well as serially after TIL infusion (days 7, 21, 28, and 42 and at the time of imaging). 70 mL of blood was drawn on all follow-up visits, when possible. Peripheral blood mononuclear cells (PBMC) were extracted from the blood samples and evaluated using flow cytometry (described later) including a tetramer analysis for MART-1-specific CD8^+^ T cells to determine whether MART-1 peptide-pulsed DC induced the expansion of detectable MART-1-specific CD8^+^ T cells after infusion. Response was assessed by physical examination and appropriate serial imaging (PET/CT or contrast-enhanced CT of the chest, abdomen, and pelvis, as well as CNS imaging with MRI or CT) at 6 weeks and 12 weeks after cell infusion. Treatment response was evaluated using irRC. Adverse event monitoring was conducted from the time of lymphodepletion initiation and through the duration of hospital stays and follow-up; it was graded using the National Cancer Institute Common Toxicity Criteria for Adverse Events CTCAE V.3.0 (NCI 2006).

### Treatment schedule

Patients randomized to the DC arm underwent leukapheresis around 1 month before TIL infusion. All patients underwent non-myeloablative, lympho-depleting intravenous chemotherapy consisting of cyclophosphamide (60 mg/kg/day) for 2 days (days −7 and −6) (where day 0=day of TIL infusion) and intravenous fludarabine (25 mg/m^2^/day) for 5 days (days −5 to −1) as inpatients before TIL infusion. Freshly harvested and washed autologous TILs were infused intravenously. Patients randomized to the DC covaccine arm received a first dose of DC infused intravenously 4 hours post-TIL infusion (range of 98–478 x 10^6^ MART-1 peptide-pulsed DC). High dose of 720 000 IU/kg intravenous IL-2 was administered every 8 hours on days 1 to 5. Doses were skipped if patients experienced grade III or IV toxicity because of high-dose IL-2, except for the reversible grade III toxicities that are common to high-dose IL-2. On day 21, the DC infusion was repeated for patients who had been randomly assigned to the DC arm. On days 22 to 26, high-dose IL-2 was given to all patients using the same procedure described for days 1 to 5 (online supplemental figure 1A).

10.1136/jitc-2021-002449.supp1 Supplementary data

### TIL expansion and reactivity assessment TIL expansion and reactivity assessment

Patients underwent resection of a metastatic melanoma tumor deposit. The tumor was minced into small fragments of 1–3 mm^3^ which were placed in culture in individual wells of a 24-well plate in media supplemented with 6000 IU/mL of IL-2 (Proleukin, Clinigen) for a period averaging between 3 and 5 weeks as previously described.^8^ Cultures reaching at least 40 million TIL within 5 weeks were deemed successful and cryopreserved for future use. An aliquot of cells was submitted for flow cytometric evaluation of the proportion of MART-1 reactive CD8^+^ T cells in successful cultures according to validated procedure of the MDACC Stem Cell Transplantation & Cellular Therapy Flow Cytometry laboratory which adheres to the College of American Pathologists standards for flow cytometry (performance and analysis). A threshold of 0.1% CD8^+^ TIL MART-1 reactivity was set as a minimum to select the culture for large scale expansion and patient treatment. Cultures meeting this requirement were used in a Rapid Expansion Protocol (REP) for large scale expansion with anti-CD3 antibody (clone OKT3, Orthoclone), irradiated pooled allogeneic normal donor PBMC feeder cells (used at a ratio of 1 TIL to 200 feeder cells), and 6000 units per ml IL-2. The REP was initiated in T175 flasks (Nunc) for the first 7 days and then transferred into 3 L cell culture bags (Baxter or OriGen) for the last 7 days as the culture expanded. All cultures were maintained under the current Good Tissue Practice and current Good Manufacturing Practice (cGMP).

### DC vaccine generation

For vaccine production, DC were generated from PBMCs that had been derived from apheresis products collected from patients prior to treatment. At least 2 weeks prior to apheresis, patients received G-CSF (Granulocyte-colony stimulating factor) for 5 days, administered on an outpatient basis (10 µg/kg/day, subcutaneously). Apheresis was performed via a two-armed approach or via a temporary central venous catheter. The freshly collected cells were used to produce monocyte derived DC according to cGMP. Briefly, the collected cells were washed and plated in T175 flasks (175×10^6^/flask) for 2 hours at 37C. Non-adherent cells were washed away while the remaining, adherent cells were incubated with GM- CSF and IL- 4 (1000 IU/mL of each) to mature monocytes into DCs. After 5–7 days of culture, immature DCs were activated overnight with a maturation cocktail (IL-1β 10 ng/mL, TNFα 10 ng/mL, IL-6 15 ng/mL and PGE2 1 µg/mL). The next day the mature DCs were harvested, washed and then pulsed with 3 µg/mL MART-1 (26- 35 (27L); ELAGIGILTV, manufactured GMP grade by Clinalfa) peptide for 1.5 hours. The peptide pulsed DCs were washed and cryopreserved in two bags for the two doses to be administered on day 0 and day 21, in freezing solution containing 7.5% DMSO, and stored in the vapor phase of liquid nitrogen. Maturation status of DC was assessed by flow cytometry for coexpression of CD11c, CD80, CD83, CD86 and HLA-DR. DC were later thawed for infusion into patients 4 hour after TIL infusion on day 0 for the first dose and 3 weeks later for the second dose (online supplemental figure 1A).

### Flow cytometry analysis on expanded TIL and PBMCs

Cryopreserved TIL or PBMCs were thawed, washed in FACS (Fluorescence Activated Cell Sorting) Wash Buffer [1X Dulbecco’s phosphate buffered saline (PBS) with 1% Bovine Serum Albumin]and stained for cell surface markers; CD8 PB (clone RPA-T8, BD (BD Biosciences)), MART-1 dextramer APC (Immudex), CCR7 PerCP-Cy5.5 (clone G043H7 BioLegend), CD45RA FITC (clone HI100, BD), CD27 APC-H7 (clone M-T271, BD), CD28 PE-Cy7 (clone CD28.2, BD), CD3 PE (clone SK7, BD). A viability stain was included for dead cell exclusion (the fixable dye Live/dead stain-Aqua, Invitrogen, or 7-AAD (7-Aminoactinomycin D) for fresh stains). The cells were fixed and acquired using a BD FACS Canto II flow cytometer. Live cells were gated based on fluorescence minus one controls. The data were analyzed using Flow Jo software (BD).

### Immunohistochemical analysis

Formalin-fixed paraffin-embedded tissue (FFPE) specimens were obtained from melanoma patients who underwent a tumor harvest for TIL expansion, using a portion of the same tumor lesion that was provided for TIL expansion. FFPE specimens were assessed by immunohistochemical analysis with a MART-1 specific antibody by the MDACC clinical pathology laboratory.

### Nanostring TCR clonotype evaluation and cloning of MART-1 TCR

TCR alpha/beta repertoires were determined by nanoString analysis of TIL whole RNA extracts. PCR amplifications of TCR alpha and beta chains from TIL cDNA were performed using subgroup variable region-specific forward primers and constant region reverse primers. As specifically described in this study, TRAV12-2 (TATTCTGGGAAAAGCCCTGA) and TRBV4-3 (AGCCACTGGAGCTCATGTTT) forward primers were used with corresponding constant region reverse primers TCR-CA (TCAGGCAGTGACAAGCAGCAATAAGGGAAC) and TCR-CB2 (CTGGGATGGTTTTGGAGCTA), respectively. Complementarity-determining regions (CDR) were identified by sequence analysis using the International ImMunoGeneTics Information System (http://www.imgt.org) V-QUEST tool.

### Statistical analysis

Wilcoxon rank-sum tests were used to compare the distribution of continuous variables between treatment arms. Fisher’s exact tests were used to compare the distribution of categorical variables between arms. The Kaplan and Meier method was used to estimate the distribution of overall survival (OS) and progression-free survival (PFS) from the treatment date. Patients who were lost to follow-up were censored at their last contact date. Distributions were compared among arms using the log-rank test. Statistical analyses for survival were performed using R software V.3.6.1. All statistical tests used a significance level of 5%. No adjustments were made for multiple testing. Statistical analysis of the T-cell attributes was performed using GraphPad Prism V.8.4.3. Groups were compared using an unpaired two tailed Mann-Whitney U test and p values under 0.05 were considered significant.

## Results

### Patient characteristics and treatments

Trial enrolment was divided into two turnstiles. Turnstile I constituted in the tumor harvest for small scale TIL expansion.^2 8^ All patients were HLA typed at MDACC HLA typing laboratory. Only HLA-A0201 positive patients whose tumor was positive for the expression of MART-1 at the protein level, and whose TIL contained at least 0.1% MART-1 reactive CD8^+^ T cells qualified to enroll on turnstile II, to proceed with large scale TIL expansion and infusion. The requirement to meet these parameters greatly restrained the number of eligible patients for the trial. Initially, the trial was designed to enroll 48 patients in the randomized cohort. Because of slow accrual, it was stopped after 23 patients. Multiple factors contributed to the slow accrual. First, the patients needed to be HLA-A0201^+^, which comprise around 50% of the Caucasian population but since our patient population is multiethnic, the rates of HLA-A0201^+^ patients were lower. Of the patients who were HLA-A0201^+^, only about half had a MART-1 recognizing TIL population in their expanded pre-REP TIL. From October 2008 to December 2012, twenty-three patients were randomized to TIL (n=12) or TIL +DC (n=11), 18 of whom received therapy. Two patients from TIL arm and three patients from TIL +DC arm did not receive study drug as planned and were excluded from analysis. In the TIL arm, one patient withdrew consent and another patient died before the initiation of treatment. In the TIL +DC arm, two patients experienced rapidly progressive disease (PD) and leptomeningeal disease; because of time constraints and the length of time it would take for TIL to initiate, the physician chose to start other treatments. Another patient received one cycle of TIL but was found to be ineligible for analysis as a result of concurrent BRAF inhibitor treatment. All randomized patients’ characteristics are shown in online supplemental table 1. Therefore, data from a total of 18 treated patients were evaluated and analyzed in the study.

All evaluated patients’ characteristics are described in table 1. Most patients had advanced disease; 16 (89%) had stage M1c, including 10 (56%) with brain metastasis, and 3 (17%) had high LDH (lactate dehydrogenase) levels. Sixteen patients (89%) had undergone at least one prior systemic treatment, including targeted therapy, immunotherapy, or chemotherapy, whereas two patients (11%) were treated as first-line therapy for metastatic melanoma. Almost all enrolled patients were checkpoint naïve at time of treatment (17/18) except for one patient who had been treated with a checkpoint inhibitor (10 mg/kg ipilimumab) prior to TIL therapy (patient #448); 16 months had passed from the last ipilimumab administration before TIL harvest and TIL ACT therapy. Presence of MART-1 positive tumor cells was confirmed in all patients.

**Table 1:** Patient characteristics

Characteristic	All (n=18)	TILs (n=10)	TIL+DC (n=8)	P value
Median age (range), years	43 (18–64)	49 (32–63)	38 (18–64)	0.2
Sex				
Female	9	4	5	0.6
Male	9	6	3	
White race	18	10	8	–
Melanoma type				
Cutaneous	14	8	6	0.8
Acral	1	0	1	
Undetermined	3	2	1	
Stage				
4 M1b	2	0	2	0.2
4 M1C	16	10	6	
ECOG*				
0	9	2	7	**0.02**
1	9	8	1	
LDH				
Normal	15	8	7	1.0
High	3	2	1	
BRAF mutation				
BRAF V600 E	10	7	3	0.3
BRAF non-V600 E	2	2	0	
WT	4	1	3	
NRAS mutation				
Q61K	3	1	2	0.5
WT	9	6	3	
KIT mutation				
c-KIT	1	1	0	1.0
WT	11	7	4	
Prior systemic therapies				
None	2	0	2	0.07
1	7	3	4	
2	7	6	1	
3	1	1	0	
4	1	0	1	
No of infused TILs (range)	60.7 (1.7–130)	38.2 (1.7–99)	83.2 (16.3–130)	**0.04**
No of doses of IL-2 infused	14 (3–17)	14 (11–17)	12.5 (3–17)	0.3

*ECOG status: 0=asymptomatic, 1=symptomatic and ambulatory.

CR, complete response; DC, dendritic cells; ECOG, Eastern Cooperative Oncology Group; IL-2, interleukin-2; irRC, immune-related response criteria; PD, progressive disease; PR, partial response; SD, stable disease; TIL, tumor-infiltrating lympocytes; WT, wild type.

The study arms were well balanced for patients’ demographic characteristics and treatment attributes (online supplemental table 1), except for the total TIL infused and the baseline ECOG performance status. The number of TIL infused was higher in the TIL+DC arm than in the TIL alone arm (median, 83.3 vs 38.2, p=0.04); patients in the TIL alone arm were more likely to have an ECOG score of 1 than were patients in the TIL+DC arm (p=0.02).

An overview of the infused cellular products for each patient is shown in table 2. In the TIL+DC arm, seven patients received two DC vaccines and one patient received one DC vaccine. The patient did not receive the second DC dose due to death.

**Table 2:** Overview of infused cellular products

Patient #	Cohort	No of DC vaccines	Total no of injected DC per vaccine (x10^6^)	No of infused TILs (billions)	Total no of IL-2 doses	%CD8	%CD8 Mart-1+	Clinical response*	OS† Months	PFS months
126	TIL	0	0	8	14	22.7	6.94	SD	8**‡**	5
228	TIL	0	0	74	11	80.4	0.072	PD	129	2
232	TIL	0	0	99	17	95.4	64.7	PD	86	4
307	TIL	0	0	46	18	91.7	4.95	PR	49**‡**	11
351	TIL	0	0	32.6	15	18.7	0.16	PD	14**‡**	2
408	TIL	0	0	69	14	80.5	6.25	SD	83	4
411	TIL	0	0	14.3	11	38.5	1.31	PD	22**‡**	1
420	TIL	0	0	43.8	14	13.7	1.83	SD	15	3
448	TIL	0	0	27.3	12	35.8	0.13	CR	53**‡**	18
456	TIL	0	0	1.7	17	56.9	–	PR	11**‡**	2
120	TIL+DC	2	250	44.6	17	18	3.01	SD	17**‡**	6
151	TIL+DC	2	184	62	14	76.4	0.72	SD	102	5
172	TIL+DC	2	209	115	15	81.4	0.82	CR	137	–
177	TIL+DC	1§	478	130	3	97.2	0.28	PD	2**‡**	0
302	TIL+DC	2	201	104.5	10	95.1	96.6	PR	7**‡**	7
312	TIL+DC	2	328	107	12	66.3	1.63	PD	119	1
380	TIL+DC	2	250	16.3	13	4.68	17.8	PR	17**‡**	8
412	TIL+DC	2	98	55	12	72	–	PR	31**‡**	7

*Best overall response measured by irRC.

†As of September 2020.

‡Deceased.

§Patient died before second DC dose.

ACT, adoptive cell therapy; CR, complete response; DCs, dendritic cells; ORR, overall response rate; OS, overall survival; PD, progressive disease; PFS, progression free survival; PR, partial response; SD, stable disease; TILs, tumor-infiltrating lymphocytes.

### Immunomonitoring and functional ex vivo analysis

**Figure 1:**
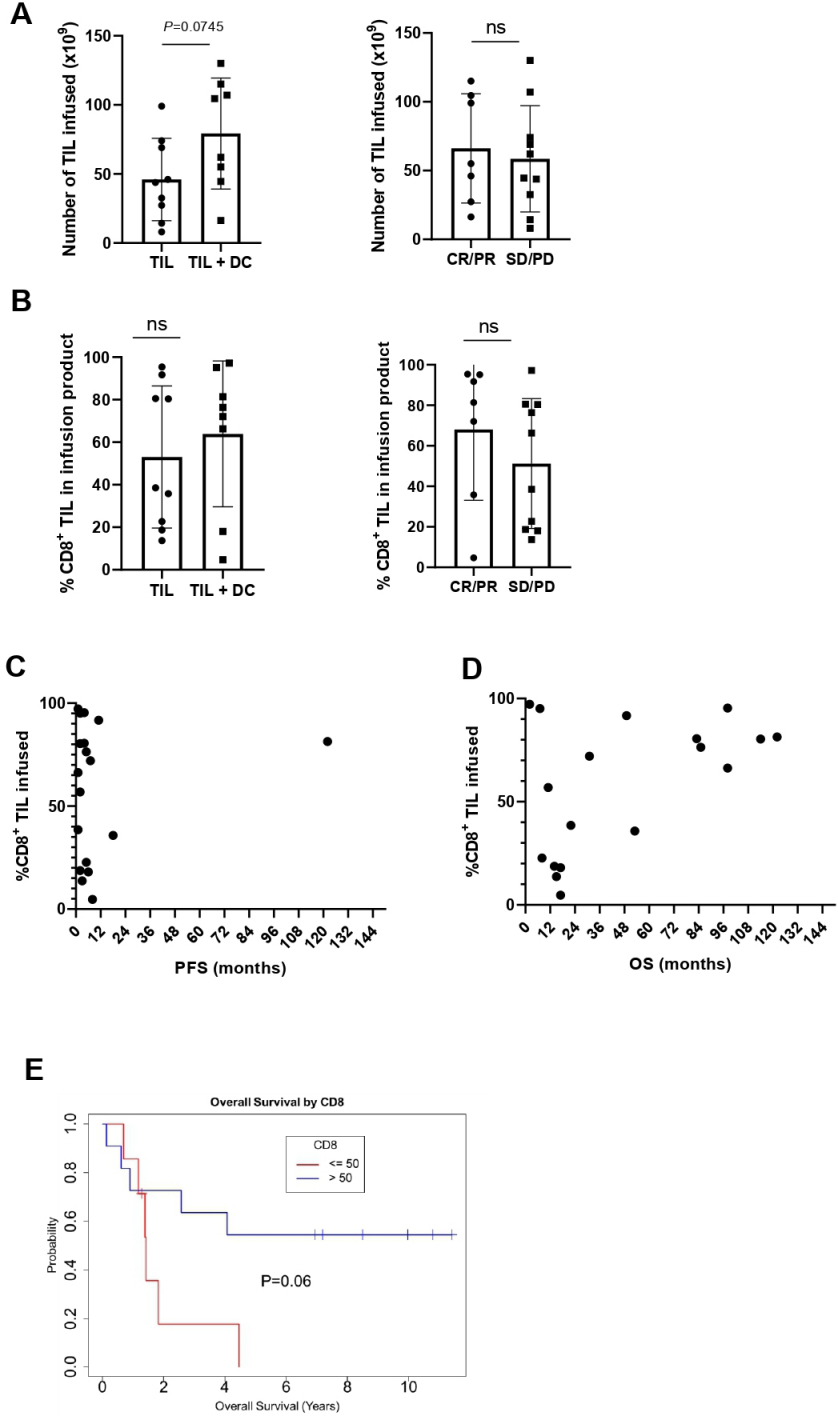
The infusion of a larger proportion of CD8^+^ TIL tends to associate with longer survival. (A) Total number of TIL infused by arm (left panel) or responsecircles represent patients treated with TIL alone while squares denote patients who received TIL +DC. Mean and SD are shown. Statistics were calculated with double sided Mann-Whitney U test. (B) %CD8^+^ TIL infused by arm (left panel) or response (right panel). Circles represent patients who responded (CR or PR) while squares denote patients who did not respond (SD or PD). Mean and SD are shown. Statistics were calculated with double sided Mann-Whitney U test. (C) %CD8^+^ TIL infused and PFS D) %CD8^+^ TIL infused and OS. (E) Kaplan-Meier plot of OS stratified by %CD8^+^ TIL infused (≥50% or <50%). CR, complete response; DC, dendritic cell; ns, not significant; OS, overall survival; PFS, progression-free survival; PD, progressive disease; PR, partial response; SD, stable disease; TIL, tumor-infiltrating lymphocytes.

In prior studies, we have found an association between the number of TIL infused and the percentage of CD8^+^ TIL in the infusion product, with outcome to TIL therapy in metastatic melanoma.^2 8^ In this study, patients treated on the TIL +DC arm received a higher number of TIL on average (p=0.04, figure 1A). However, there was no correlation between the number of TIL infused and response to TIL therapy (figure 1A). The percentage of CD8^+^ TIL infused was not significantly different between patients on the TIL or TIL +DC cohorts, additionally the percentage of CD8^+^ TIL was also not significantly associated with clinical response in the overall cohort (figure 1B). Nonetheless, the patient who experienced a very long PFS of over 90 months had a majority (>50%) of CD8^+^ TIL in its infusion product (figure 1C), and patients who lived longer (OS over 5 years) were infused with a TIL product containing a high proportion of CD8^+^ TIL (figure 1D). Overall patients who had >50% CD8+TIL in their infusion product had a trend toward a longer OS (p=0.06, figure 1E).

**Figure 2:**
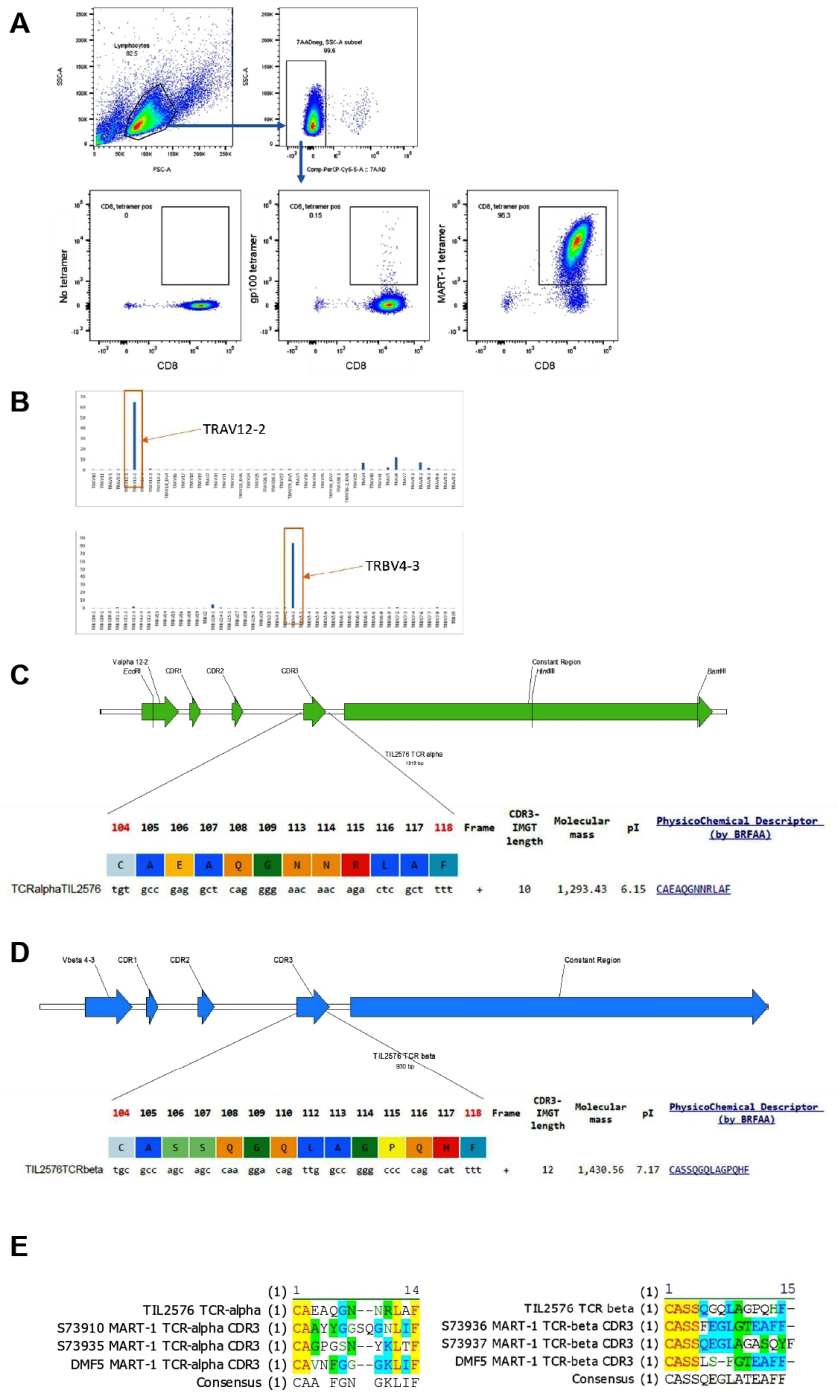
MART-1 reactivity of TIL #302 infusion product. (A) Flow cytometry evaluation of MART-1 reactivity in infusion product from TIL #302. Gating strategy demonstrates the gating of viable (7AAD negative) lymphocytic population. The cells were stained for CD8 and either no tetramer (left plot), gp100 dextramer (middle plot) or MART-1 dextramer (right plot). (B) Nanostring analysis of TCR clonotypes. (C) Sequencing data of the CDR3 region of the alpha chain of the TCR. (D) Sequencing data of the CDR3 region of the beta chain of the TCR. (E) Alignment of the CDR3 sequence of the alpha and beta chains of the TCR with published sequences from CDR3 regions of MART-1 recognizing TCRs. MART-1, melanoma antigen recognized by T cells 1; TIL, tumor-infiltrating lymphocytes.

Two patients had a short OS after being infused with a TIL product containing a high frequency of CD8^+^ TIL (TIL #302, OS=7 months and TIL #177, OS=1.5 months; figure 1D). The overall response rate (ORR) of TIL #302 was partial response (PR) and of TIL #177 was PD. Of note, TIL #302, was retrospectively found to have been infused with a large number of a clonal population of MART-1 reactive TIL. Flow cytometry performed on the infusion product revealed that the product was 95.1% CD8^+^, and that 96.6% of those CD8^+^ recognized MART-1 antigen (figure 2A). NanoString analysis of RNA extracted from the infusion product of this patient investigating TCR alpha chain and beta chain diversity demonstrated a strong enrichment for one TCR alpha chain (TRAV12-2) and one TCR beta chain (TRBV4-3) (figure 2B).^27^ Cloning of the dominant TCR followed by sequencing of the CDR3 regions of the alpha chain and beta chain revealed a unique CDR3 region for each, demonstrating that the population was clonal (figure 2C, D). Alignment of the TCR alpha and beta CDR3 region sequence with other published MART-1 specific HLA-A0201 restricted TCR sequences revealed conserved motifs within the beta CDR3 regions^31 32^ (figure 2E). Review of the clinical history of the patient revealed that he was enrolled on a tumor vaccine protocol and was vaccinated with a MART-1 peptide prior to enrolling on the TIL therapy trial. The pretreatment tumor of this patient was found to be 60% positive for MART-1 antigen by clinical pathology.

### Analysis of MART-1 specific TIL clones in infusion product and PBMCs

**Figure 3:**
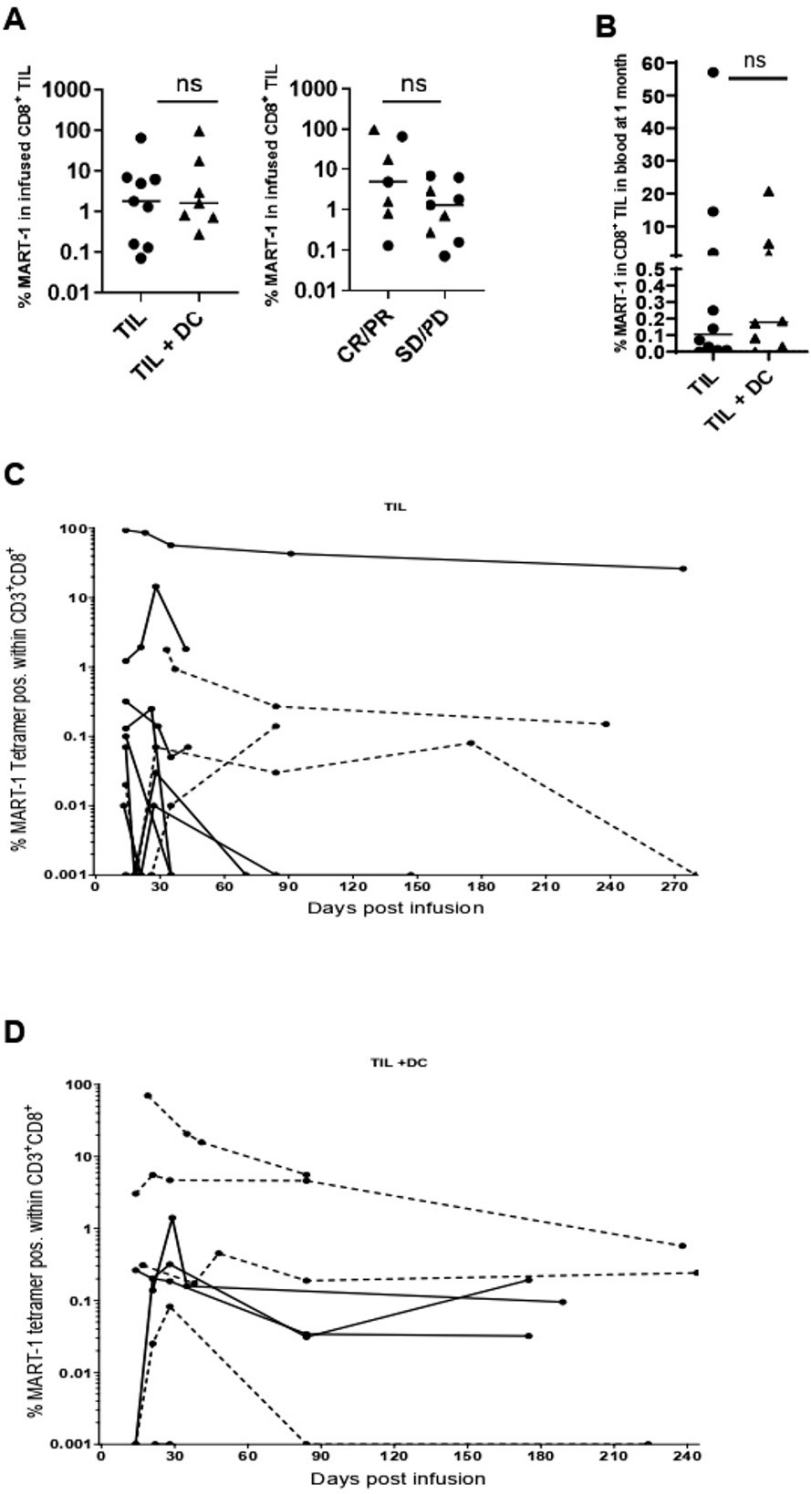
Persistence of MART-1 reactive CD8^+^ TIL after infusion. (A) %MART-1 reactive CD8^+^ TIL infused by arm (left panel) and response (right panel). Circles represent patients treated with TIL alone while triangles denote patients who received TIL+DC. Statistics were calculated with double-sided Mann-Whitney U test. (B) %MART-1 reactive CD8^+^ TIL, as a proportion of total CD8^+^ T cells, circulating in the blood at 1 month post TIL infusion, by arm. Circles represent patients treated with TIL alone while triangles denote patients who received TIL+DC. Statistics were calculated with double sided Mann-Whitney U test. (C) Longitudinal tracking of MART-1 reactive CD8^+^ T cells in the blood of patients treated in the TIL arm. Dotted lines indicate Responder patients. Values less than 0.001% (or undetected) were represented as 0.001% to allow visualization on a logarithmic axis (D) longitudinal tracking of MART-1 reactive CD8^+^ T cells in the blood of patients treated in the TIL+DC arm by flow cytometry. Dotted lines indicate Responder patients. values less than 0.001% (or undetected) were represented as 0.001% to allow visualization on a logarithmic axis. DC, dendritic cell; ns, not significant; MART-1, melanoma antigen recognized by T cells 1; TIL, tumor-infiltrating lymphocytes.

The proportion of MART-1 reactive CD8^+^ TIL in the final product varied widely (0.07% to 96.6%) but was not significantly different between patients in the TIL or TIL+DC arms or between responders and non-responders (figure 3A). We next investigated if the combination of TIL+DC pulsed with MART-1 peptide leads to increased persistence of MART-1 reactive TIL. The frequency of MART-1 reactive TIL was measured by flow cytometry in longitudinal blood samples. No difference was observed in the frequency of MART-1 specific CD8^+^ T cells between patients in the TIL or TIL+DC arms at 30 days postinfusion (figure 3B). Longitudinal tracking of the MART-1 reactive T cells in the blood postinfusion showed that persistent MART-1 reactive T cells could be detected in patients from both arms. (figure 3C, D).

Because there was no difference in persistence of the MART-1 reactive T cells between arms, the study did not meet its primary endpoint.

### Clinical efficacy

**Figure 4:**
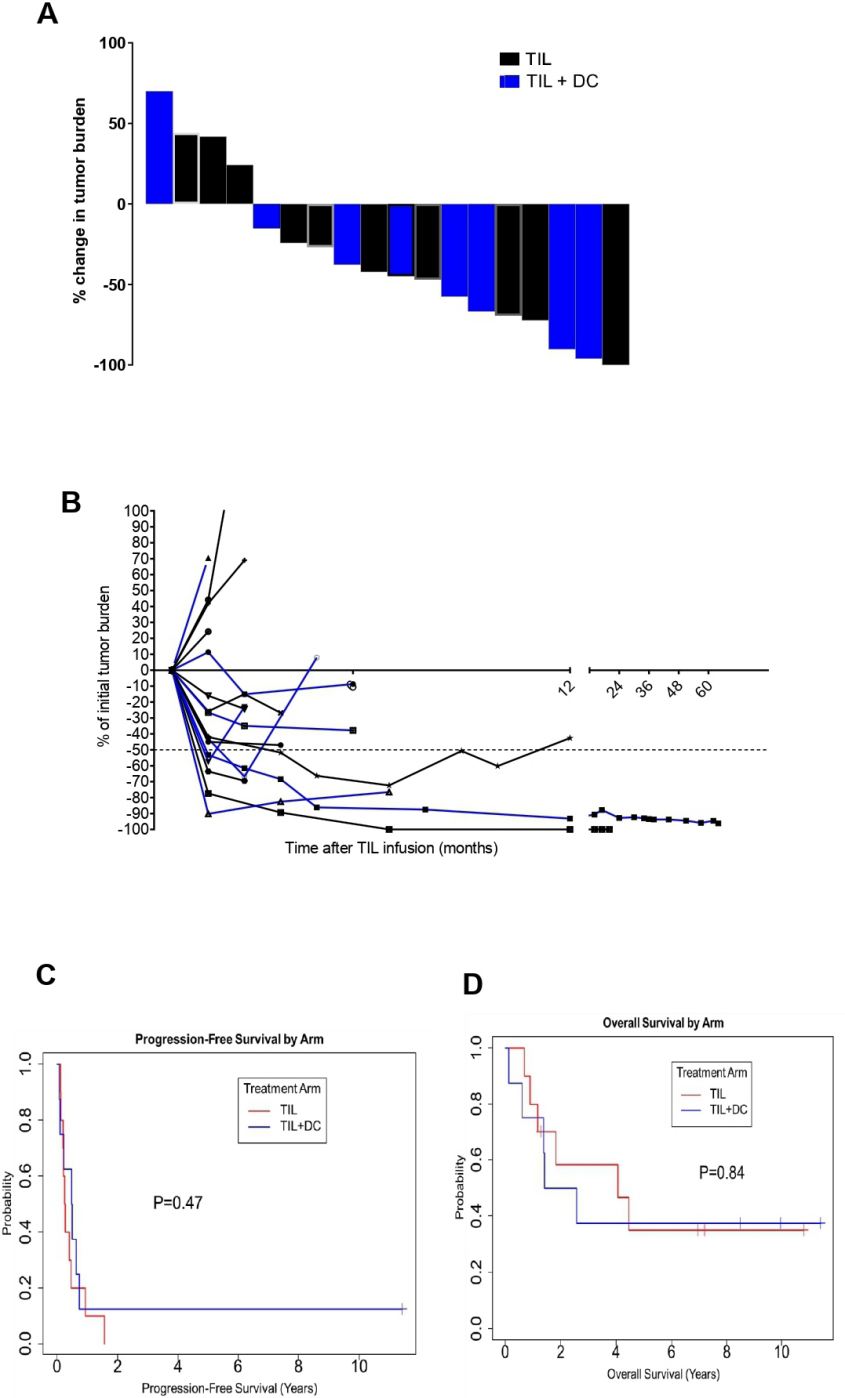
Clinical response to TIL with or without DC co-vaccination. (A) Waterfall plot (B) spider plot depicting the tumor burden of every patient at every time point collected. Patients in the TIL arm are denoted by a black curve while patients in the TIL +DC arm are denoted by a blue curve. (C) Kaplan-Meier plot showing pfs by treatment arm. (red curve is for TIL alone and blue curve is for TIL+DC patients). The median PFS duration was 0.26 years in the TIL arm and 0.49 years in the TIL +DC arm. The 2-year PFS rate was 0% (95% CI 1.6% to 64%) in the TIL arm and 13% in the TIL +DC arm (95% CI 13% to 78%). (D) Kaplan-Meier plot of os by treatment arm. (Red curve is for TIL alone and blue curve is for TIL +DC patients). The median OS duration was 4.1 years in the TIL arm and 2.0 years in the TIL+DC arm. at 2 years, the OS rate was 58% (95% CI 34% to 100%) in the TIL arm and 50% in the TIL +DC arm (95% CI 25% to 100%). DC, dendritic cell; OS, overall survival; PFS, progression-free survival; TIL, tumor-infiltrating lymphocytes.

The ORR was 30% for the 23 enrolled patients and was 39% for the 18 treated and evaluated patients. By treatment arm, in 23 randomized patients, the ORR was 25% in TIL alone arm and was 36% in the TIL+DC arm. In the 18 treated and evaluated patients, the ORR was numerically higher in the TIL+DC arm (4/8; 50%) compared with the TIL arm (3/10; 30%), although this did not reach statistical significance. A waterfall plot of the best overall response for the treated and evaluated patients is presented in figure 4A and a spider plot depicting the kinetic of tumor burden is shown in figure 4B. In the TIL arm, two patients experienced a PR (TIL #307 had a duration of 11 months, and TIL#456 had a duration of 2 months), and one patient (TIL#420) experienced a CR that lasted for 18 months. The remaining patients in the TIL arm had stable disease (SD) for <4 months or PD (three SD and three PD) (table 2). In the TIL+DC arm, one patient achieved a CR (TIL#172, duration of >137 months; was ongoing at last follow-up) and three patients achieved PRs; the three PRs had a response duration of 2, 7 and 8 months. The remaining four patients either had SD for <5 months or PD (two SD and two PD) (table 2). The median duration of response was 0.76 years in the TIL arm (95% CI 0.09 to 1.4 years) and was 0.81 years (95% CI 0.09 to 11.2 years) in the TIL+DC arm (online supplemental figure 2). Responses were ongoing in one of four (25%) patients in the TIL+DC arm and none of three (0%) patients in the TIL arm.

With a median follow-up of 2.2 years (range, 0.13–10.22 years), the median OS duration was 2.6 years (95% CI CI 01.4-NA years) and the median PFS duration was 0.34 years (95% CI 0.21 to 0.74 years) in the whole cohort (online supplemental figure 3A, B, respectively). The PFS duration was longer in the TIL +DC arm than in the TIL arm (0.49 years and 0.26 years, respectively) (p=0.47) (figure 4C). The OS duration was longer in the TIL arm than in the TIL +DC arm. (4.1 years and 2 years, respectively), but this difference did not reach statistical significance (p=0.84) (figure 4D).

**Figure 5:**
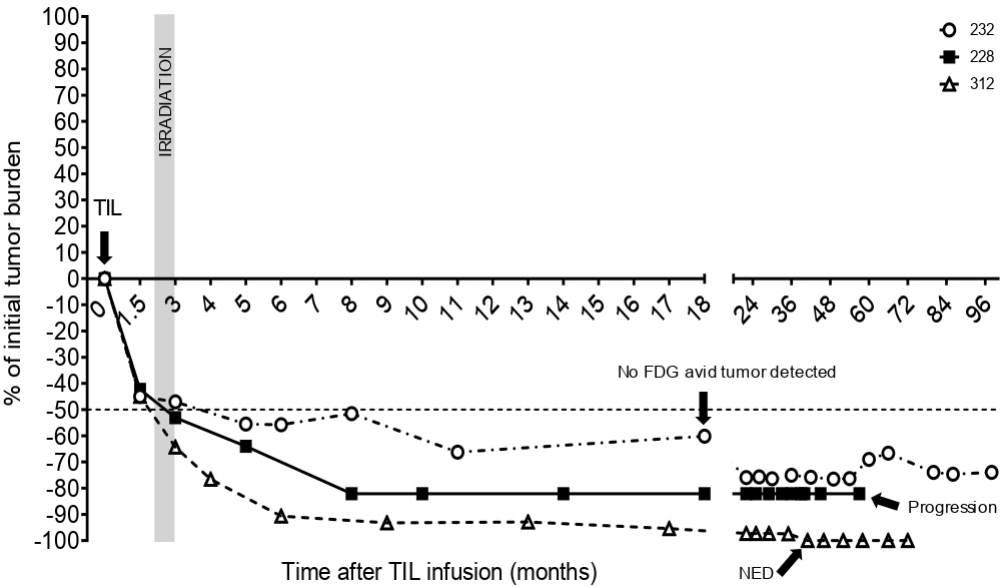
Long-term systemic tumor control following local radiation of the brain in patients who had received TIL. Systemic tumor burden decreases over time in three patients who received local intracranial radiation of the brain within a month of being treated with TIL therapy (Patients #228, 232 and 312). TIL, tumor-infiltrating lymphocytes. Abbreviations: FDG, Fluorodeoxyglucose. NED, No evidence of disease.

Following progression, nine patients died without further intervention, four patients received immunotherapy and targeted therapy (TIL #307, #351, #408, and #151), one patient was lost to follow-up (TIL #420), and three patients received stereotactic radiosurgery (SRS) for management of CNS metastases (TIL #228, #232, and #312). Interestingly in these three patients we observed long term systemic tumor control following intracranial radiation of brain metastasis. Following radiation, they not only experienced intracranial response but they also experienced rapid and continued decrease of their extracranial lesions (located in the liver for TIL#228 and in the lungs for TIL#232 and TIL#312) without further progression of disease or any intervention, with ongoing responses lasting for 5 years (TIL #228), over 8 years (still ongoing, TIL #232), and over 10 years (still ongoing, TIL #312) (figure 5A).

### Treatment-related toxicities

The incidence and grade of each TIL-related adverse event are summarized in online supplemental table 3). There were no grade 5 adverse events. All patients experienced the expected hematological grade 2–3 toxicities with lympho-depleting chemotherapy, which were reversable with blood and platelet transfusions and G-CSF growth factors. They also experienced toxicities caused by high-dose IL-2 therapy that resolved by 8–10 days after cell infusion, except for two patients (TIL#177 and #351): one experienced reaction to TIL infusion, with fever, chills, and peripheral edema, and the other experienced grade 2 and grade 3 capillary leak syndrome, respectively. The DC vaccine was well tolerated in this trial; only two patients experienced grade 1–2 adverse events, including injection site pain after the first DC vaccine injection. No other grade 1–2-adverse events or any grade 3–4 adverse events were observed.

Immune-related adverse events targeting MART-1 expressing in normal cells were noted in one complete responder patient (TIL#172) who developed vitiligo after receiving TIL+DC. In the TIL arm, one patient (TIL#232) developed blurry vision 6 days following TIL infusion, which resolved with an eye drop hydration; uveitis was not confirmed by the ophthalmologist.

## Discussion

In metastatic melanoma patients receiving ACT using TIL, the persistence of infused TIL as well as the enrichment of TIL for certain tumor specificities have been shown to correlate with better clinical response.^6 23^ We sought to prolong the persistence of CD8^+^ TIL recognizing the tumor antigen MART-1 by covaccinating with autologous DCs presenting the MART-1 antigen. Here we show that MART-1 reactive TIL durably persist after infusion but their persistence was not significantly changed by the infusion of two doses of MART-1 peptide-pulsed autologous DC vaccine.

The trial design was developed based on our prior mouse modeling demonstrating that DC covaccination with adoptive transfer of antitumor T cells provides superior tumor control.^24^ In this setting, the treatment of melanoma tumor bearing mice with the adoptive transfer of transgenic T cells recognizing the gp100 antigen with concomitant intravenous infusion of gp100-pulsed bone-marrow derived DCs resulted in improved tumor control and T-cell persistence as compared with the mice treated with T cells only. All transferred T cells in this system recognize the antigen pulsed on the DCs, which is uniformly expressed by the tumor. Our translation of this approach to patients demonstrated activity of this regimen, although we did not observe a significantly improved persistence of the MART-1 recognizing TIL infused. There are several differences between the mouse model and the clinical trial. For example, the design of the clinical trial involved DC infusion 4 hours after TIL infusion, which is different than the mouse studies where DC were infused immediately after T cells. It is possible that within 2 hours after infusion a large proportion of the TIL localized to the lungs, spleen, and liver and were not available to be restimulated by the DC.^33^ Other key differences include the source of the DCs (bone marrow derived, or monocyte derived), the DC maturation method, and the number of target antigen-specific T cells in the infusion product (very variable in patients, and as low as 0.1%, but 100% in mouse model). Finally, probably the most important difference is the status of differentiation of the antitumor T cells used, as mouse models use peripherally (spleen or lymph nodes) derived T cells which contain a large fraction of naïve T cells whereas human TIL are effector memory cells with high expression of checkpoint molecules from chronic stimulation.

Recently, another group reported sustained responses in 4/4 patients with advanced melanoma receiving TIL +DC vaccine, where the DC were pulsed with a tumor lysate, which would lead to presentation of multiple tumor antigens, and potentially direct reactivation of a larger repertoire of infused TIL.^30^ In this phase I trial, TIL infusion was followed by five doses of DC, with an intention to restimulate injected TIL with the DC vaccine. This approach has the added advantage of not being restricted to a particular HLA type or a specific tumor antigen, factors that complicated enrollment on our study. Moreover, this other study administered DCs intradermally while our study used intravenous infusion of DCs. Data from mouse modeling has suggested superiority of the intradermal DC infusion over intravenous in reactivating tumor-specific T cells ultimately leading to better tumor control.^34 35^ There is evidence in melanoma patients that intradermally administered DCs can efficiently re-stimulate T cells.^35 36^ Overall, our study found good persistence of the MART-1 specific TIL after infusion in both arms of the trial, which may be confounded by the fact that TIL in general persist at high levels after TIL therapy but may also suggest that the intravenous DC vaccination performed in this study did not optimally re-stimulate the MART-1 specific TIL. Further testing of the best route of administration for DC covaccine to optimally support TIL activation in patients is warranted.

The clinical response was numerically higher in TIL+DC arm (4/8, 50%) compared with TIL arm (3/10, 30%), although this study was not powered to detect statistical significance between the two arms. Previous studies have shown that high numbers of TIL infused, and especially high number of CD8^+^ TIL, correlate with favorable response.^2 8^ Our study arms were not balanced for this confounding factor since the patients were randomized before TIL expansion and since the magnitude of TIL expansion is highly variable between patients. The numbers of TIL infused was higher in the TIL+DC arm than in the TIL alone arm (p=0.04). Hence, the underlying reason for slightly higher clinical response is uncertain and could be attributed to higher number of TIL infused in the combination arm. Due to the small number of enrolled patients, no definitive conclusion on efficacy between the arms was possible.

TIL therapy alone has proven effective for metastatic melanoma but the efficacy of TIL in the context of the checkpoint-refractory patient population is yet unclear. Our earlier study reported lower clinical response among melanoma patients who received TIL ACT after prior exposure to anti-CTLA4 therapy compared with checkpoint naïve patients in a cohort of 74 patients.^2^ However, two other studies showed that previous exposure to anti-CTLA4 has no impact on response to ACT TIL.^9 10^ Emerging data suggest that response to TIL therapy after progression on anti-PD-1 is reduced to the lower to 32%–36% range.^37 38^ In our study, patients were treated between 2008 and 2012 which was before anti-PD-1 approval and only one patient received ICI prior to TIL therapy in the form of ipilimumab, therefore we could not address the question of prior checkpoint exposure on TIL response. Since checkpoint blockade has become the standard of care for metastatic melanoma, further studies are needed to evaluate and compare the impact of prior exposure to immune checkpoint inhibitors on response to TIL ACT.

One of our infused patients was treated with over 100 billion of a highly clonal MART-1 reactive CD8^+^TIL population. This patient saw a rapid tumor burden reduction followed by loss of tumor control and death within 7 months. Pathology evaluation of the baseline levels of MART-1 expression in the tumor indicated 60% positivity rate within the tumor cells. Unfortunately, we do not have any tumor biopsies following progression to investigate the correlation between disease progression and the level of tumor MART-1 expression following progression for this patient or any other patient on the trial. It is possible that after rapid clearance of the MART-1 positive tumor cell population the rest of the tumor cell mass started growing unchecked by TIL. This case highlights the limitations of single antigen targeting in solid tumor cellular therapy.

MART-1 is a tumor-associated melanocytic differentiation antigen that is overexpressed in most metastatic melanoma but also expressed in normal melanocytes.^39^ The prevalence of T cells recognizing MART-1 antigen is unusually high in the peripheral blood of HLA-A0201^+^ normal donors and melanoma patients. This high frequency has been linked to a biased usage of TRAV12–2 variable gene of the TCRα-chain, as well as to the lack of expression of the complete MART-1 transcript in the thymus, causing a lack of central tolerance against T cells recognizing this epitope from a normal protein.^40^ MART-1 has been studied as a model tumor antigen in melanoma for several decades. Transferred MART-1 reactive TIL have been shown to be able to persist at high levels in the blood for extended period (>150 days), maintaining cytotoxic antitumor function.^41^ Because of the high likelihood of the presence of MART-1 specific TIL in the tumors of HLA-A0201^+^ melanoma patients and its demonstrated value as tumor antigen we chose MART-1 as antigen for our study. However, based on the data showing that MART-1 specific TIL may persist in the blood at high frequency after infusion independently of DC help, in retrospect MART-1 may not have been the optimal antigen to use for our study. Other tumor antigens for which there is a lower TIL frequency may have benefited more from a DC covaccine approach. The limitation we faced is that there are no other known universally shared antigens in melanoma with demonstrated tumor rejection potential that we could feel reasonably confident to find TIL reactivity to in most patients enrolled in the study.

One patient who experienced a CR (ongoing for >10.22 years) in the TIL+DC arm, developed widespread vitiligo on the forearm, face, and perineal area that was noted 5 months after the second DC dose. This vitiligo is assumed to be caused by MART-1 specific targeting of normal skin cells by T cells, which could be enhanced by DC vaccination Hence, the observed autoimmunity represents on-target, off-tumor killing, which demonstrates efficient targeting or MART-1 antigen in vivo, suggesting that MART-1 targeting of the tumor may also have played a role in preventing relapse of melanoma in this patient. Of note, autoimmune-like manifestations such as vitiligo or uveitis have been reported following TIL therapy where the infused TIL contained a population of MART-1 reactive CD8^+^ TIL and have been associated with clinical response.^41^

Recently various studies showed that local radiation therapy (RT) could stimulate the systemic antitumor immune response.^42 43^ The proposed mechanism is that the apoptotic and necrotic tumor bed following radiation release tumor associated antigens, which are presented to the CD8^+^ cytotoxic T cells by the DCs. Activated immune cells may migrate to sites of disease throughout the body providing systemic tumor control.^43^ In our study, we observed that local RT used in conjunction with TIL led to prolonged tumor control. Three patients, who progressed into the brain within 1 month of TIL therapy, received SRS immediately (post 1 month following TIL therapy) for their intracranial lesions. Interestingly, the three patients experienced not only intracranial tumor response but also continued systemic tumor shrinkage over many years without further intervention. Based on this observation in our study, one could speculate that radiation led to antigen release from dying cancer cells, cross-presentation on DCs, with a resulting enhancement of TIL activities, suggesting that the combination of radiotherapy and ACT with TIL can result in synergistic antitumor responses. This is an intriguing observation that should be investigated in further studies using combination strategies of local RT and ACT with TIL in patients with advanced melanoma.

## Conclusions

Our study did not demonstrate that the addition of a DC vaccine to TIL therapy enhances MART-1 TIL persistence. Long-term persistence of infused MART-1 reactive TIL was measured in select patients from both arms. The lack of benefit of DC covaccination may be attributable to the antigen chosen since MART-1 reactive TIL naturally occur at high frequency in melanoma patients and had been previously shown to persist after infusion without the help of a DC vaccine. The trial was not powered to resolve differences in clinical response rate between the two arms. Our data suggest that further testing of the addition of a DC vaccine targeting a single tumor antigen to the TIL therapy regimen may not be warranted, however, it is worth noting that the clear limitation of the study is the focus on one single tumor antigen and that multiantigen DC covaccination approaches have shown early signs of efficacy and are worth pursuing.

In addition, to our knowledge, this is the first study to report that combining local RT with TIL-ACT could enhance the efficacy of TIL ACT for metastatic melanoma. This finding deserves further investigation in a larger series of patients.

## Data Availability

Data sharing not applicable as no datasets generated and/or anlayzed for this study. All data relevant to the study are included in the article or uploaded as online supplemental information.
