# Supplementary material for: Randomized phase II trial of lymphodepletion plus adoptive cell transfer of tumor-infiltrating lymphocytes, with or without dendritic cell vaccination, in patients with metastatic melanoma
Source: J Immunother Cancer. 2021 May 21;9(5):e002449. doi: 10.1136/jitc-2021-002449 (PMC8144048; doi:10.1136/jitc-2021-002449)
Supplement: Supplementary data [file jitc-2021-002449supp001.pdf]

Supplementary Figure 1

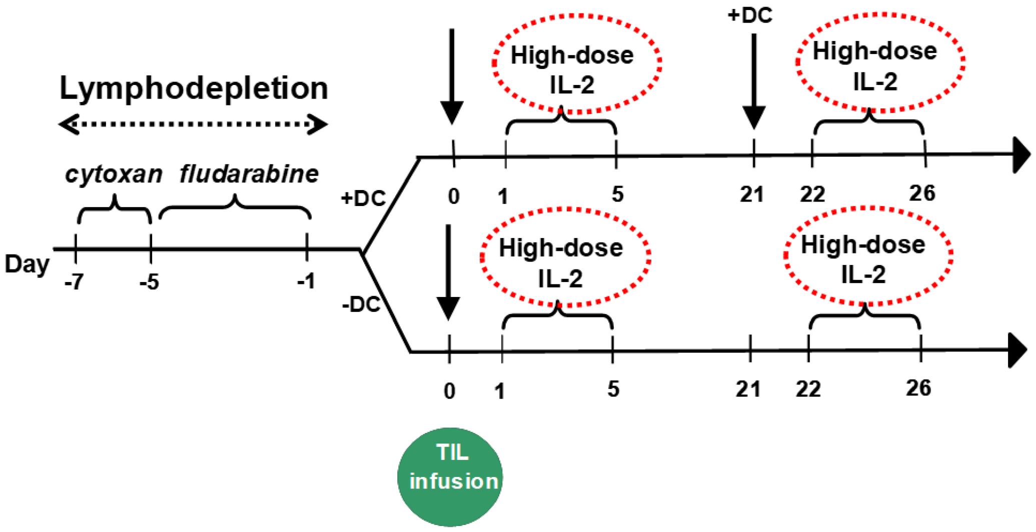

Supplementary table 1: All randomized patients Characteristics

**Characteristic**	**Enrolled (n=23)**	**TILs (n=12)**	**TIL+DC (n=11)**	**P value**
Median age (range), years	48 (18-71)	49 (32-71)	41 (18-64)	0.15
Sex				
Female	10	4	5	1.00
Male	13	6	3	
White race	23	10	8	-
Melanoma type				
Cutaneous	17	9	8	1.00
Acral	2	1	1	
Undetermined	3	2	1	
Mucosal	1	0	1	
Stage				
4 M1B	2	0	2	0.2
4 M1C	21	12	9	
ECOG*				
0	10	2	8	**0.005**
1	9	8	1	
LDH#				
Normal	15	8	7	1.0
High	4	2	2	
BRAF mutation				
BRAF V600 E	12	7	5	
BRAF non-V600 E	2	2	0	0.3
WT	4	1	3	
Unknown	5	2	3	
NRAS mutation				
Q61K	3	1	2	0.5
WT	11	7	4	
Unknown	9	4	5	
KIT mutation				
c-KIT	2	2	0	0.5
WT	12	7	5	
Unknown	9	3	6	
Number of infused TILs (range)	-	38.2 (1.7-99) †	69.4 (16.3-130) ‡	**0.03**

Abbreviations: DC, dendritic cells; ECOG, Eastern Cooperative Oncology Group (0=asymptomatic, 1=symptomatic and ambulatory); WT, wild type; TIL, tumor-infiltrating lymphocytes.

*In four patients, the ECOG was not evaluated
#In four patients, the LDH level was not measured
†Two patients did not receive any TIL therapy; one patient withdrew consent and another patient died before the initiation of treatment
‡ Two patients did not receive any TIL + DC therapy due to rapidly progressive disease. One patient received only one cycle of TIL + DC but was found to be ineligible for analysis as a result of concurrent BRAF inhibitor treatment.

BMJ Publishing Group Limited (BMJ) disclaims all liability and responsibility arising from any reliance placed on this supplemental material which has been supplied by the author(s)

Supplementary Figure 2.

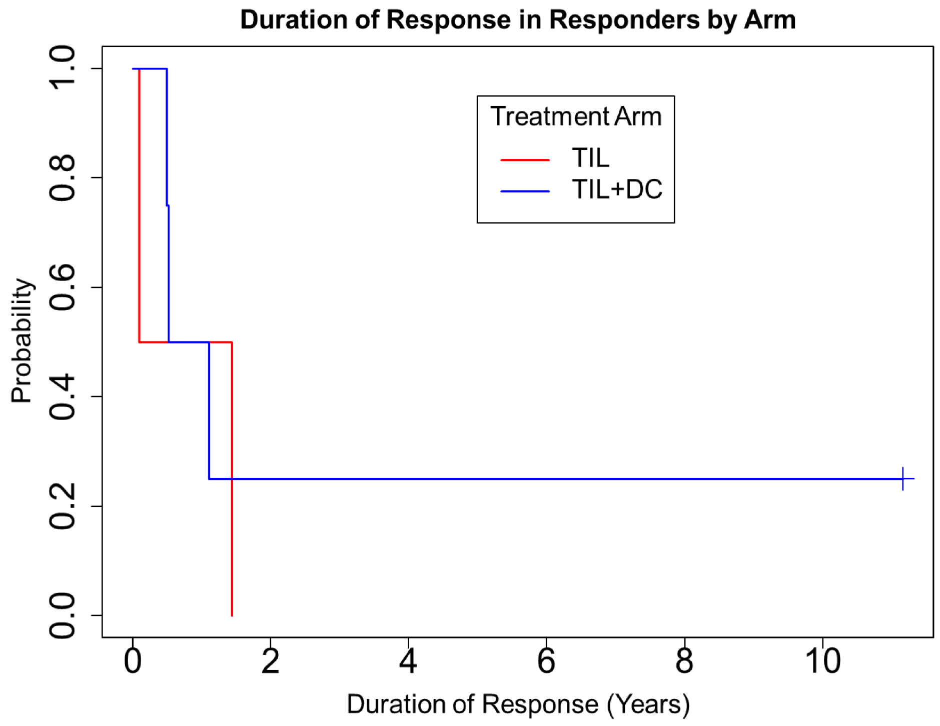

Supplementary Figure 3.

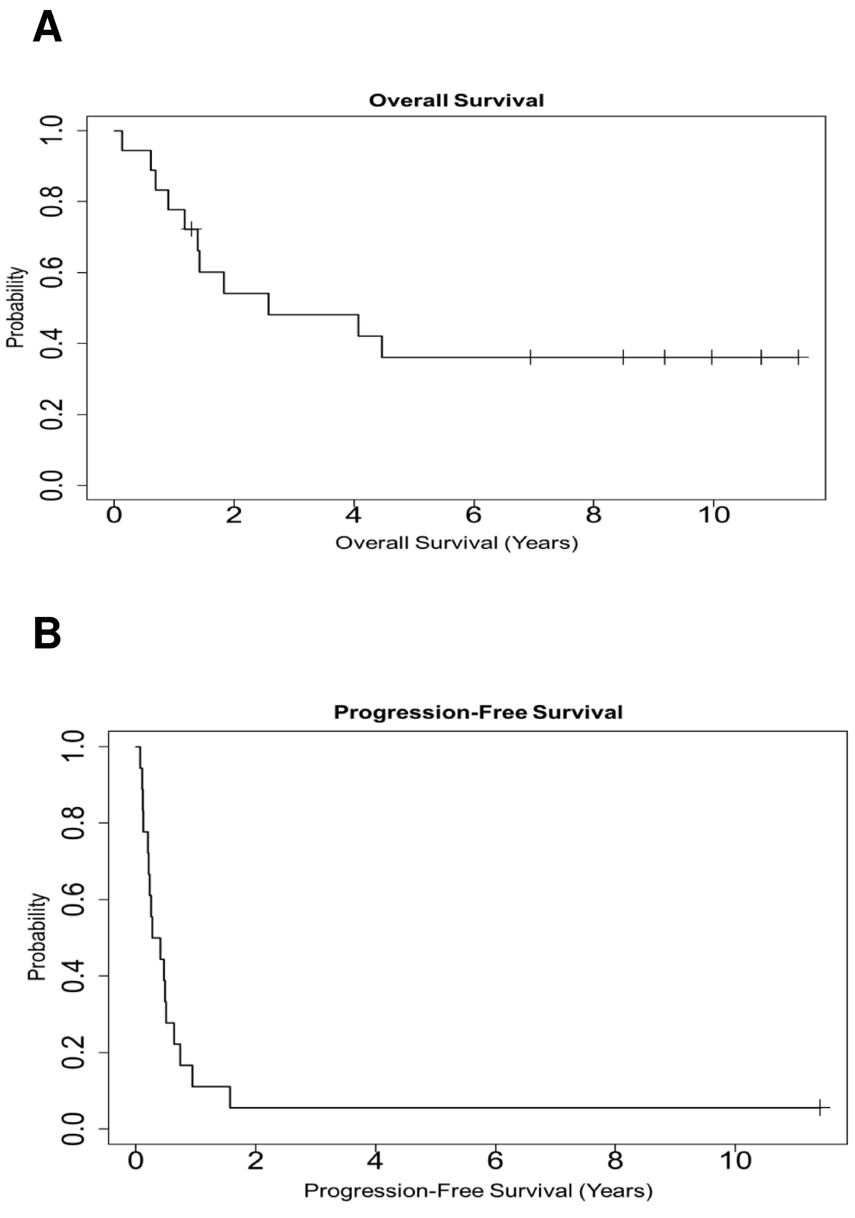

Supplementary Table 1. Adverse events related to treatment

	Maximum grade, number			
**Toxicities**	**1**	**2**	**3**	**4**
Hematologic				
Neutropenia		4	6	
Thrombocytopenia		4	6	
Leukopenia		9	1	
Lymphopenia	1	3		
Anemia	3	3	4	
Gastrointestinal				
Nausea/vomiting	4	1	1	
Diarrhea	2			
Constipation	1	1		
Anorexia	2	1	1	
LFT increase	6			
Bilirubin	2	1		
Blood in stool and melena	2			
Renal				
Electrolyte imbalance	6	3	1	
AKI creatinine	5		1	
Urine output decreased	1	2		
Proteinuria	1			
Neurologic				
Fatigue	4	3		
Mental status changes	1			
Headache	1			
Change in vision	1			
Dermatologic				
Alopecia	1	1		
Dry eyes	1			
Phlebitis		1		
Rash	3	2		
Pruritis	3	1		
Vitiligo		1		
Vascular leak syndrome				
Edema, weight gain	8	3		
Dyspnea	4	2		
Capillary leak syndrome	1	1	1	
Pleural effusion	2			
Hypotension	1	1		
Fever/chills	3	2		

AKI, acute kidney injury; LFT, liver function tests
